# Supplementary material for: Identification of enriched hyperthermophilic microbial communities from a deep-sea hydrothermal vent chimney under electrolithoautotrophic culture conditions
Source: Sci Rep. 2021 Jul 20;11:14782. doi: 10.1038/s41598-021-94135-2 (PMC8292307; doi:10.1038/s41598-021-94135-2)
Supplement: Supplementary file 1 — Supplementary Information. [file 41598_2021_94135_MOESM1_ESM.pdf]

Identification of enriched hyperthermophilic microbial communities from a deep-sea hydrothermal vent chimney under electrolithoautotrophic culture conditions.

Guillaume Pillot[1], Oulfat Amin Ali[1], Sylvain Davidson[1], Laetitia Shintu[3], Anne Godfroy[2], Yannick Combet-Blanc[1], Patricia Bonin[1], Pierre-Pol Liebgott[1*]

[1] Aix Marseille Univ., Université de Toulon, IRD, CNRS, MIO UM 110, 13288, Marseille, France

[2] IFREMER, CNRS, Université de Bretagne Occidentale, Laboratoire de Microbiologie des Environnements Extrêmes – UMR6197, Ifremer, Centre de Brest CS10070, Plouzané, France.

[3]Aix Marseille Univ, CNRS, Centrale Marseille, iSm2, Marseille, France

[*] To whom correspondence may be addressed. Email: pierre-pol.liebgott@mio.osupytheas.fr; Mediterranean Institute of Oceanography, Campus de Luminy, Bâtiment OCEANOMED, 13288 Marseille Cedex 09.

https://orcid.org/0000-0002-2559-1738

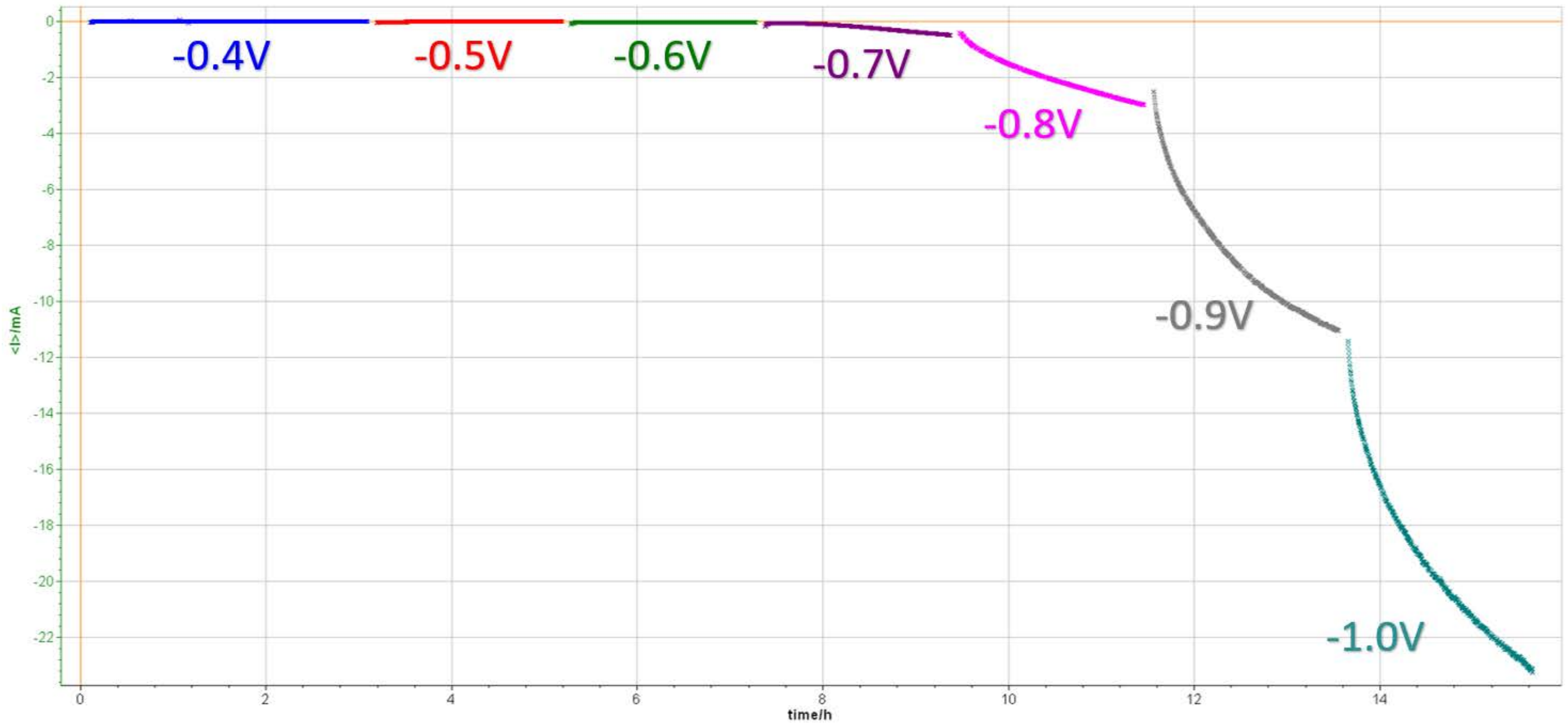

Supplementary Figure S1: Screening of potentials in abiotic and anaerobic conditions to select the lowest potential before water electrolysis. Potentials are expressed vs. SHE.

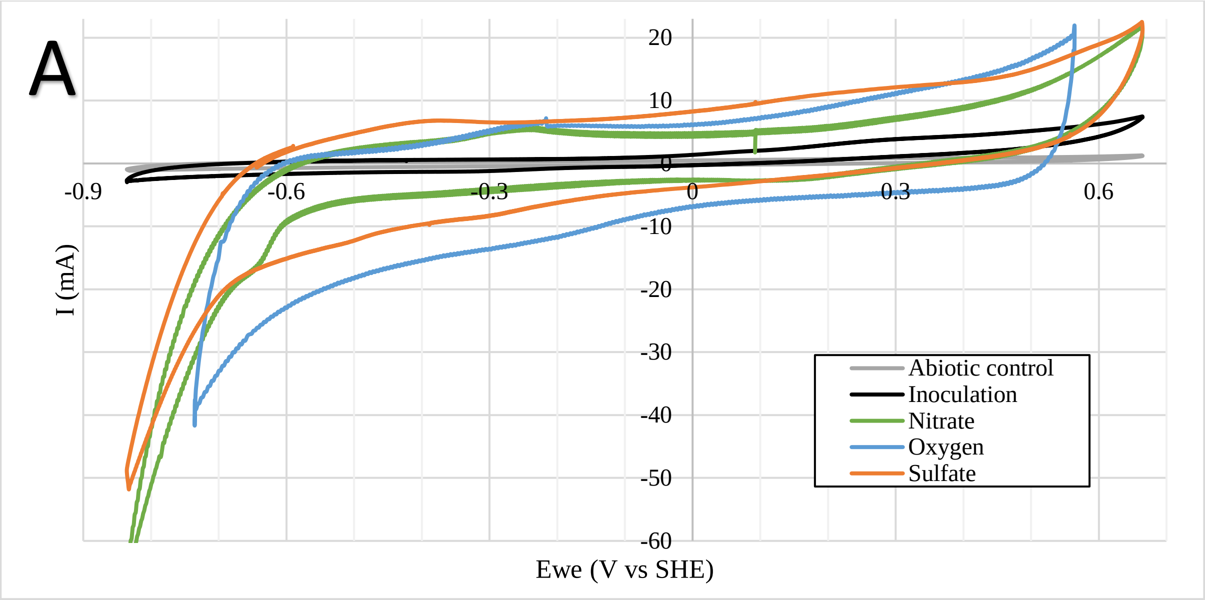

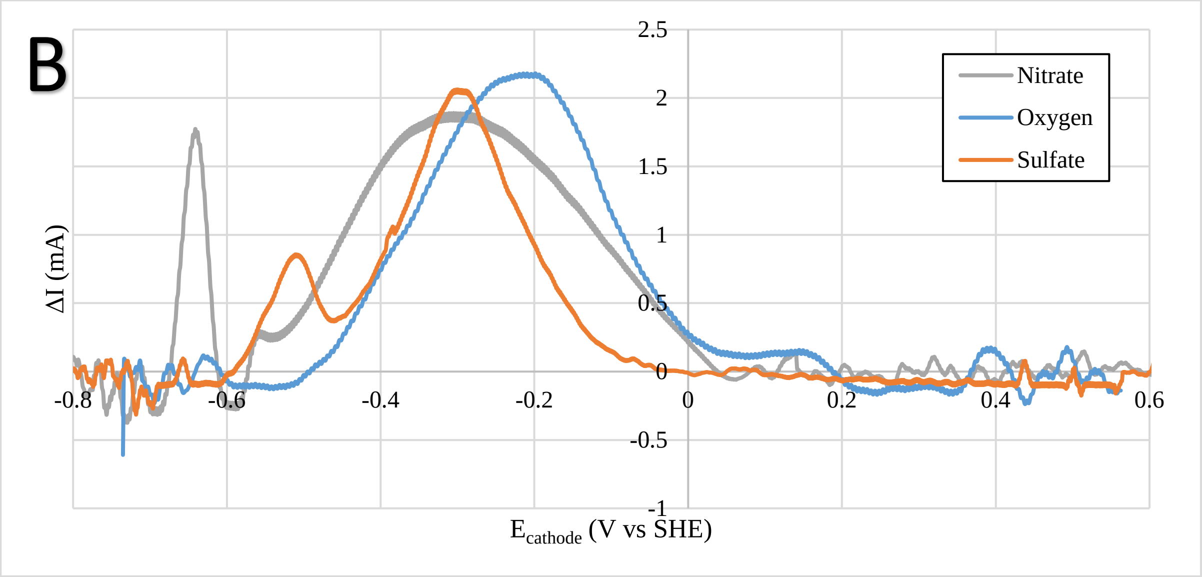

Supplementary Figure S2: A) Cyclic Voltammograms (scan rate = 20 mV/s) of the abiotic control, and of the experiments at inoculation time and after 30 days for each condition (Nitrate, Oxygen and Sulfate). B) Reduction peaks extracted from Cyclic Voltammograms (scan rate = 20 mV/s) where the baseline have been subtracted with the software QSoas. The ΔI of reduction peaks are expressed in inversed values. Cyclovoltammetries carried out with a 3 M Ag/AgCl reference electrode (E= +0.165 V vs SHE at 80°C).

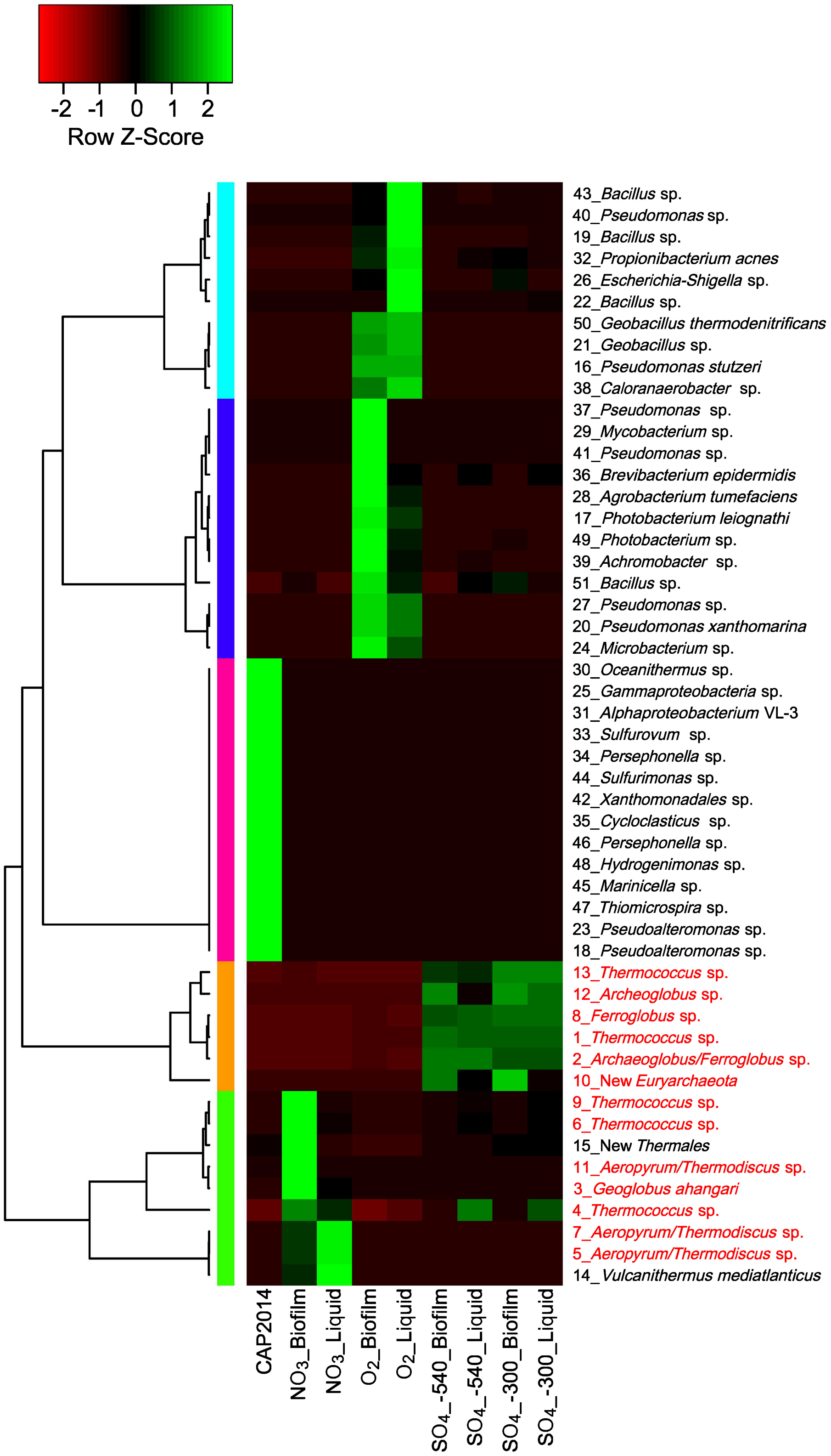

Supplementary Figure S3: Heatmap representation of the distribution of dominant OTUs (>0.5%) over the different electron acceptors (Liquid: Liquid Media/planktonic cells). OTUs and samples clustering were performed with centroid average method and with Pearson distance measurement method. The red taxa represent the *Archaea* members and black taxa, the *Bacteria*. RStudio: Integrated Development for R. RStudio, PBC, Boston, MA URL http://www.rstudio.com/ and the package gplots by Warnes et al. (2020).

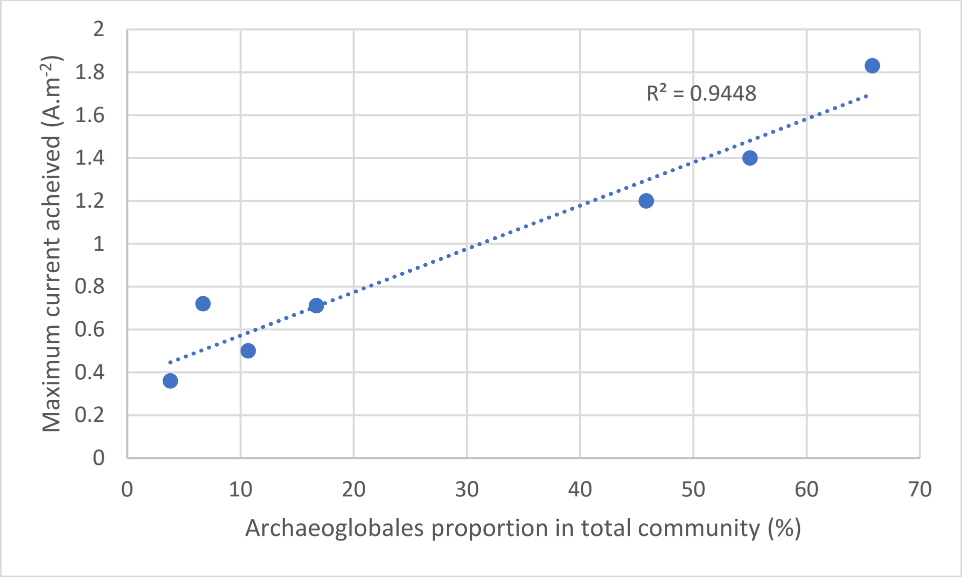

Supplementary Figure S4: Correlation between maximum current and proportion of *Archaeoglobales* in the total community measured by Metabarcoding and weighted by the total qPCR quantification. The data were obtained from the 4 enrichments presented in this study, 2 subcultures from Nitrate enrichment and one enrichment on Fe(III)Oxide enrichment, not presented in this study.

	Time (days)														
**Transient compounds**	**0**	**1**	**2**	**3**	**4**	**5**	**7**	**9**	**11**	**12**	**13**	**15**	**16**	**18**	**21**
Benzoate like	5.6	0.0	29.4	19.8	24.2	25.5	54.4	55.0	76.6	76.2	94.8	103.4	116.1	99.0	128.4
Methanol	3.2	0.0	0.0	10.8	16.1	31.5	9.0	36.0	9.2	0.0	0.0	7.2	0.0	4.6	5.4
Formate	1.9	61.5	8.4	54.9	21.8	70.5	23.4	63.0	25.8	17.0	32.6	23.6	0.0	13.4	22.0
Cystine	12.3	35.5	30.2	23.7	30.6	23.6	17.7	13.8	9.6	7.8	1.2	2.6	0.0	0.0	19.4
Acetoacetate	0.0	0.0	0.0	0.0	0.0	0.0	0.0	44.0	4.6	15.4	0.0	0.0	0.0	0.0	0.0
Lactate	6.6	30.0	24.0	15.0	10.6	31.5	9.2	37.0	11.0	12.2	17.2	16.0	27.6	11.0	13.0
Threonine	1.9	11.5	2.6	3.6	4.0	3.0	4.2	0.0	2.2	2.0	0.0	2.0	8.7	1.2	4.0
Succinate	1.3	0.0	2.2	0.0	0.0	0.0	0.0	0.0	0.0	0.0	0.0	0.0	0.0	0.0	1.2
Ethanol	2.2	177.0	40.6	37.5	42.5	47.5	29.8	86.0	16.8	0.0	33.6	0.0	57.9	0.0	6.4
Alanine	0.0	0.0	0.0	10.8	12.0	10.5	13.8	0.0	20.2	0.0	54.2	93.6	178.5	179.8	236.6
Acetamide	0.0	0.0	0.0	0.0	20.0	25.5	48.6	54.0	57.6	69.2	39.4	35.8	18.9	10.6	6.4
2-Aminoisobutyric acid	4.8	67.0	0.0	5.1	4.2	6.5	15.2	28.0	19.6	21.6	12.0	13.2	8.1	0.0	0.0
3-hydroxyisovalerate	4.6	37.5	7.6	28.2	6.6	34.5	9.4	81.0	13.8	8.0	14.4	11.2	23.4	9.8	14.6

Supplementary Table S1: Evolution of transient compounds (in µM) over the enrichment on Nitrate measured by NMR.

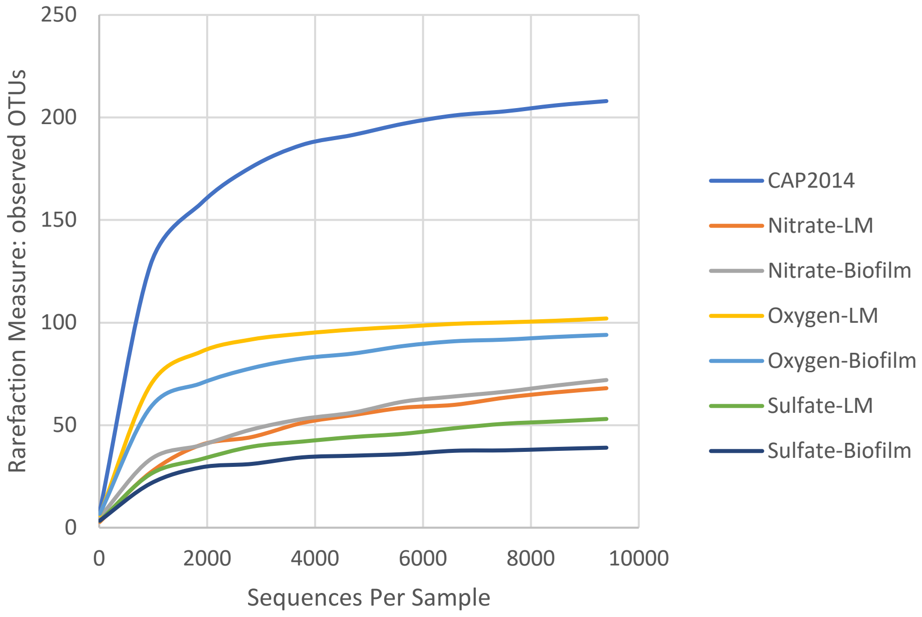

Supplementary Figure S5: Rarefaction curves of 16S rDNA sequences for bacterial and archaeal diversities in the different samples. LM: Liquid media; Biofilm (cathode). Curves were calculated on OTUs at 97% similarity.
